# Identification of faecal extracellular vesicles as novel biomarkers for the non‐invasive diagnosis and prognosis of colorectal cancer

**DOI:** 10.1002/jev2.12300

**Published:** 2023-01-05

**Authors:** Zhaowei Zhang, Xuehui Liu, Xiaoqing Yang, Ying Jiang, Ang Li, Jiying Cong, Yuwei Li, Qinjian Xie, Chen Xu, Dingbin Liu

**Affiliations:** ^1^ State Key Laboratory of Medicinal Chemical Biology Research Center for Analytical Sciences and Tianjin Key Laboratory of Molecular Recognition and Biosensing College of Chemistry Nankai University Tianjin China; ^2^ Tianjin Institute of Urology the 2nd Hospital of Tianjin Medical University Tianjin China; ^3^ Department of Colorectal Surgery Tianjin Union Medical Center Tianjin Institute of Coloproctology School of Medicine Nankai University Tianjin China; ^4^ Gansu Corps Hospital of CAPF Lanzhou China; ^5^ College of Pharmacy Third Military Medical University Chongqing China

**Keywords:** biomarkers, clinical diagnosis, colorectal cancer, extracellular vesicles, faeces

## Abstract

Colorectal cancer (CRC) is one of the most common malignancies that is usually detected late in the clinic. The currently available diagnostic tools for CRC are either invasive or insensitive to early lesions due to the dearth of reliable biomarkers. In this study, we discovered that the extracellular vesicles (EVs) in the faeces of CRC patients can act as a potent biomarker for the non‐invasive diagnosis and prognosis of CRC. This finding is based on the identification of two transmembrane proteins—CD147 and A33—on faeces‐derived EVs (fEVs) that are intrinsically associated with CRC. The detection results show that the levels of CD147 and A33 on fEVs were upregulated in the CRC patients (*n* = 48), dramatically distinguishing them from the healthy donors (*n* = 16). The CD147/A33‐enriched EVs offer a clinical sensitivity of 89%, much higher than that (40%) of carcinoembryonic antigen (CEA), a clinically‐established serum biomarker for CRC diagnosis. In addition, the analysis of longitudinal faeces samples (*n* = 29) demonstrated that the CD147/A33‐enriched fEVs can be utilized to track the prognosis of CRC. Due to the high compliance of faeces‐based detection, the CD147/A33‐enriched fEVs could serve as new‐generation CRC biomarkers for large‐scale, non‐invasive CRC screening as well as real‐time monitoring of patient outcomes during clinical interventions.

## 1 INTRODUCTION

Colorectal cancer (CRC) ranks third in common malignancies and is the second leading cause of death concerning cancer (Sung et al., 2021). Most CRCs develop from precursor lesions such as adenomatous polyps or sessile serrated lesions (Erichsen et al., 2016). It usually takes a long time (10–20 years) to achieve cancerization, thus providing much access to detect precursor lesions and early cancer (Keum & Giovannucci, 2019). With the assistance of early‐stage cancer detection and surgical resection of lesions, CRC‐caused mortality could be effectively reduced. It was estimated that the 5‐year relative survival rate of CRC patients dramatically declines from ∼ 90% in stages I and II to <20% in stages III and IV (Crosby et al., 2022). Therefore, it is of great significance to conduct early CRC screening among the age‐appropriate population with appropriate diagnostic methods.

Currently, colonoscopy is the gold standard for CRC diagnosis and has been generally used for CRC screening. However, colonoscopic diagnosis is intensely invasive, resulting in poor compliance among subject participants (Shaukat & Levin, 2022). Moreover, the accuracy of colonoscopic diagnosis largely depends on the operator's proficiency and experience (Garborg et al., 2013). Carcinoembryonic antigen (CEA) is a well‐established serum marker for clinically diagnosing CRC, monitoring treatment, and identifying recurrence (Primrose et al., 2014). Nevertheless, it is still limited in sensitivity and specificity because the level of CEA also upregulates in other malignancies such as breast, lung, and gastric cancers (Feng et al., 2017; Grunnet & Sorensen, 2012; Tang et al., 2016). Faecal immunochemical test (FIT) (Chiang et al., 2011) and multitarget stool DNA (mstDNA) test (Cooper et al., 2018; Cotter et al., 2017; Imperiale et al., 2014), which respectively detect the haemoglobin and DNA mutations in faeces samples, represent two typical non‐invasive methods for CRC screening. However, they frequently deliver false‐positive results caused by other diseases. To make faecal samples useful for CRC diagnosis, it is of urgent demand to discover new biomarkers with high clinical sensitivity and specificity.

**FIGURE 1:**
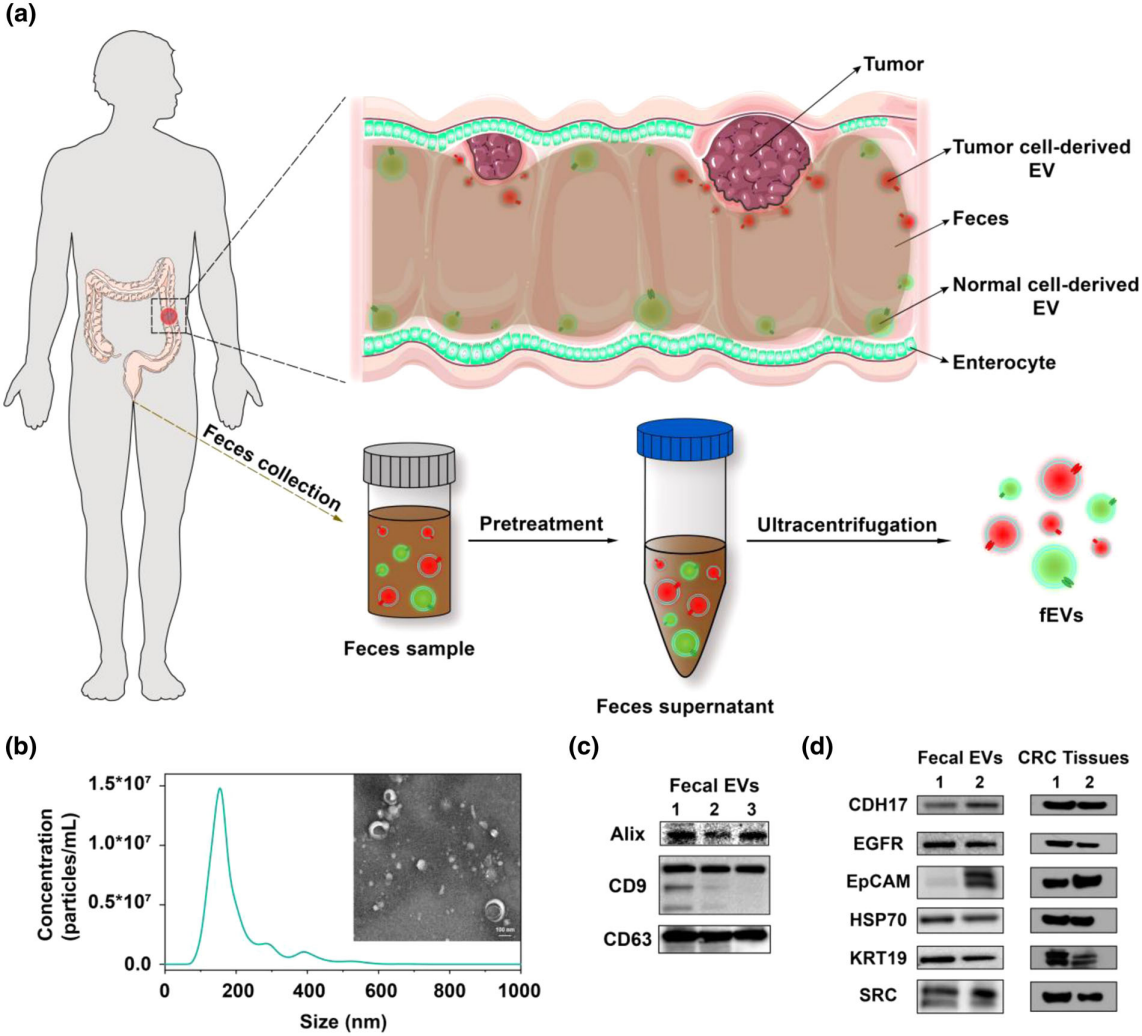
Isolation and characterization of fEVs. (a) Scheme of biogenesis and isolation of fEVs. (b) Particle size distribution of fEVs by NTA and representative morphology of fEVs by cryo‐TEM. (c) Western blotting analysis for EV general markers of fEVs collected from three CRC patients. (d) Western blotting analysis of CRC‐related proteins in the fEVs and tumour tissues collected from two patients.

Extracellular vesicles (EVs) are nano‐ and micro‐vesicles secreted by cells and released into body fluids such as serum, urine, and saliva (Hu et al., 2021; Iwai et al., 2016; Merchant et al., 2017). EVs inherit the specific molecular information from their parental cells and are thus regarded as promising cancer biomarkers for “liquid biopsy” (Shah et al., 2018; Yu et al., 2022; Zhou et al., 2020). Plasma EVs have been broadly applied for cancer diagnosis and prognosis (Liu et al., 2022; Park et al., 2021; Whiteside, 2020). Since blood circulates throughout the body, plasma EVs are derived from diverse cells and tissues (Alberro et al., 2021). As a result, the limited amount of EVs secreted by lesions are routinely submerged by the enormous number of healthy EVs in plasma. Compared with blood in circulation, body fluids in direct contact with lesions could include more pathologically relevant EVs. For instance, the urinary and salivary EVs exhibit higher sensitivity and specificity than the corresponding plasma EVs in the diagnosis of kidney (Alvarez et al., 2012) and oral diseases (He et al., 2020), respectively. We hypothesized that fEVs have a great potential as valuable biomarkers for diagnosing CRC because of the unique association between faeces and the intestinal tract (Figure 1A), similar to the relationships between urine and kidney as well as saliva and mouth. This hypothesis motivated us to find fEV biomarkers for CRC diagnosis and prognosis (the workflow is shown in Supplementary Figure S1).

## 2 MATERIALS AND METHODS

### 2.1 Materials and reagents

Detailed information on antibodies was listed in Supplementary Table S4. Phosphate buffered saline (PBS), Tween‐20, RIPA lysis buffer, phenylmethylsulfonyl fluoride (PMSF), phosphotungstic acid negative stain solution (2%), and glutaraldehyde (2.5%, EM Grade) were obtained from Solarbio (Beijing, China). Methanol was obtained from Concord Technology (Tianjin, China). Sodium dodecyl sulfate (SDS), BCA Protein Assay Kit, SDS‐PAGE sample loading buffer, prestained colour protein marker, nitrocellulose membrane, polyvinylidene difluoride membrane, skim milk powder, and BeyoECL Plus were obtained from Beyotime Biotechnology (Shanghai, China). Precast Protein Improve Gels (4%–20%, 10 wells) were obtained from Yeasen (Shanghai, China). The coating buffer was obtained from BioLegend (California, USA). Casein blocking buffer (1%) was obtained from Leagene Biotechnology (Beijing, China). Tris(hydroxymethyl) aminoethane, 2‐[4‐(2‐hydroxyethyl)‐1‐piperazinyl]ethanesulfonic acid (HEPES), ethylene diamine tetraacetic acid (EDTA), and glycine was obtained from Aladdin (Shanghai, China). TMB chemiluminescence chromogenic solution and stop solution were obtained from Abcam (Cambridge, MA).

### 2.2 Collection of clinical specimens

Blood and faeces samples from CRC patients and healthy donors were collected at Tianjin People's Hospital. Colorectal carcinoma and para‐carcinoma tissues were also collected. The whole blood samples were immediately centrifuged at 1550 × g for 30 min at 4°C to obtain cell‐free plasma, followed by centrifugation at 16,800 × g for 45 min at 4°C to remove cell debris and large vesicles. The pretreated plasma, faeces, and tissue samples were stored at ‐80°C until further analysis.

### 2.3 Isolation of fEVs by ultracentrifugation

The collected faeces sample was thawed and fully dissolved in PBS. Depending on different conditions of faeces samples, the mass/volume ratios of sample/PBS vary from 1 g/5 ml to 1 g/20 ml to ensure complete dissolution. Faeces suspension was centrifuged at 3000 × g for 10 min. The supernatant was collected, and the precipitation was fully dissolved with PBS and centrifuged at 3000 × g for 10 min. The supernatant was combined with the former and filtered with quantitative filter paper to remove residues. The filtrate was centrifugated at 10,000 × g for 30 min to remove some large vesicles and treated through a 0.22 μm filter to remove microbes. The pretreated faeces supernatant was centrifuged at 150,000 × g for 2 h using an ultracentrifuge (Beckman Coulter, OptimaTM MAX‐XP, USA) with a TYPE 45 Ti swing rotor. The pellet was collected by pipetting the supernatant, resuspended in PBS, and then centrifuged at 150,000 × g for another 2 h. The precipitated EVs were resuspended in PBS for analysis. All centrifugations were performed at 4°C.

### 2.4 Characterization of fEVs

- **Morphology characterization**. The morphology of EVs was observed by a 200 kV cryo‐transmission electron microscope (Talos F200C, ThermoFisher Scientific, USA). Each EV sample was stained with 1% uranium dioxide acetate solution and dropped onto a copper grid.
- **Measurement of particle sizes**. EV particle sizes were measured by NTA using a Malvern NanoSight NS300 instrument. The samples were diluted with PBS (filtered through a sterile 0.22 μm filter) to get an appropriate concentration. Video captures for EV movements were recorded and further analyzed by NTA software (version 3.4, NanoSight) to get EV particle sizes.
- **Total protein analysis of EVs**. The protein amount of EVs was quantified by a BCA Protein Assay Kit. 200 μl of working reagent was added into 20 μl pre‐diluted sample in a 96‐well plate. After incubated at 37°C for 30 min, the absorbance of each sample was measured at 562 nm by the microplate reader. The protein amount of each sample was calculated according to a bovine serum albumin (BSA) standard curve (refer to the BCA Protein Assay Kit instructions).
- **Western blotting analysis**. The samples were lysed by RIPA buffer with protease inhibitors (PMSF, 1 mM) on ice for 30 min followed by centrifugation at 12,000 × g for 10 min at 4°C, and the protein concentration of the supernatant was quantified by BCA test. The samples were mixed with a 5× loading buffer and heated at 95°C for 10 min. After cooling to room temperature, the samples were separated by SDS polyacrylamide gel electrophoresis (SDS‐PAGE) and transferred onto a 0.45 μm nitrocellulose (NC) membrane. Subsequently, NC membranes were blocked in TBST buffer (20 mM Tris‐HCl, 150 mM NaCl, and 0.05% Tween‐20) containing 5% (w/v) skim milk at 37°C for 1 h. Then the membranes were incubated with primary antibody (diluted according to instructions) overnight at 4°C, followed by three‐time washing with TBST. HRP‐Goat anti‐rabbit IgG and HRP‐Goat anti‐mouse IgG were diluted 1000 times by TBST and used as the secondary antibody. The membranes were incubated with the corresponding secondary antibody at 37°C for 2 h, followed by three‐time washing with TBST. Finally, the blots were treated with chemiluminescent reagents (BeyoECL Plus) and imaged with Azure c600 (Azure Biosystems, USA). The quantification of signal intensity was performed using Fiji software.
- **IG‐TEM of fEVs**. fEVs isolated by ultracentrifugation at an optimal concentration were placed on 400‐mesh formvar carbon‐coated grids and allowed to absorb for 20 min. The grids were blocked by 5% BSA (PBS) and incubated with primary antibody at 4°C overnight, followed by rinsing with 0.1% BSA (PBS). As controls, some of the grids were not treated with primary antibodies. Then the grids were incubated with 10 nm‐gold labelled secondary antibody for 2 h at room temperature. After rinsing with 0.1% BSA (PBS), the grids were fixed in 2.5% glutaraldehyde for 20 min. Finally, the grids were rinsed with PBS and distilled water, followed by contrast staining using phosphotungstic acid negative stain solution (2%). The samples were viewed and photographed by a JEM‐1400 FLASH transmission electron microscope (JEOL Ltd., Japan).

### 2.5 Western blotting analysis of intestinal tissues

The tissue samples were washed three times with pre‐cooled PBS to remove blood stains. Afterwards, the tissue samples were cut into small pieces with pre‐cooled scissors and placed in a homogenate tube. Homogenate beads and lysis solution (protease inhibitor was added before use) were added for homogenization, and then the sample was lysed on ice for 30 min. After centrifugation at 12,000 rpm for 10 min at 4°C, the protein concentration of the supernatant was quantified by the BCA test. The samples were mixed with a 5× loading buffer and heated at 95°C for 10 min. After cooling to room temperature, the samples were separated by SDS‐PAGE and transferred onto a 0.45 μm polyvinylidene difluoride (PVDF) membrane. After that, PVDF membranes were blocked in TBST buffer containing 5% (w/v) skim milk at 37°C for 1 h. Then the membranes were incubated with primary antibody (diluted according to instructions) overnight at 4°C, followed by three‐time washing with TBST. HRP‐Goat anti‐rabbit IgG and HRP‐Goat anti‐mouse IgG were diluted 1000 times by TBST and used as the secondary antibody. The membranes were incubated with the corresponding secondary antibody at 37°C for 2 h, followed by three‐time washing with TBST. Finally, the blots were treated with chemiluminescent reagents and imaged.

### 2.6 Immunofluorescence staining of intestinal tissue slides

Colorectal carcinoma and para‐carcinoma tissues were fixed in 10% formalin solution and paraffin‐embedded. After deparaffinization and rehydration of the tissue, antigen retrieval was performed using EDTA antigen retrieval buffer (pH 8.0). The sections were blocked with 3% BSA in PBS at room temperature for 30 min. Phycoerythrin (PE)‐labelled antibody (anti‐CD147 and anti‐A33, 1:200 dilution) was incubated overnight at 4°C in the dark. After that, the sections were counterstained with 4′,6‐diamidio‐2‐phenylindole (DAPI) to identify nuclei. The images were captured by fluorescent microscopy.

### 2.7 Isolation of plasma EVs by ultracentrifugation

The pretreated plasma (1 ml) was thawed and diluted twice by PBS and centrifuged at 110,000 × g for 90 min using an ultracentrifuge (Beckman Coulter, OptimaTM MAX‐XP, USA) with a TLA 100.3 swing rotor. The supernatant was removed, the pellet was resuspended with PBS, and centrifuged again at 110,000 × g for 90 min. The supernatant was removed, and the precipitated EVs were resuspended in PBS for analysis.

### 2.8 Analysis of EV proteins by ELISA

The capture antibody was diluted with coating buffer to reach a final concentration of 2 μg/ml. 100 μl capture antibody was added to each microplate well and incubated at 4°C overnight. Then the solution was discarded, followed by a three‐time washing with PBST (0.2% Tween‐20 in PBS, v/v). 100 μl blocking buffer (1% casein in PBS, w/v) was added to each well and incubated at 37°C for 1 h. After discarding the blocking buffer, 100 μl EV sample (diluted with blocking buffer, 40 μg) was added and incubated at 37°C for 3 h, followed by a three‐time washing with PBST. Then 100 μl detection antibody (diluted with blocking buffer, 1.0 μg/ml for anti‐CD147 antibody and 2.0 μg/ml for anti‐A33 antibody) was added and incubated at 37°C for 1 h, followed by a three‐time washing with PBST. Subsequently, 100 μl HRP‐conjugated secondary antibody was added to each well (diluted with blocking solution, final concentration 2 μg/ml) and incubated at 37°C for 40 min. The solution was discarded, and the plate was washed five times with PBST. Finally, 100 μl TMB chemiluminescence chromogenic solution was added to each well, and 100 μl stop solution was added after 5 min dark reaction. The absorbance at 450 nm was measured by a microplate reader.

### 2.9 Statistical analysis

The difference's significance was calculated using a two‐tailed *t*‐test by GraphPad Prism (version 8.0.1). The statistical significance of the data was estimated at 95% (*P <* 0.05) CIs. We employed the logistic regression algorithm to combine CD147 and A33 into one marker by IBM SPSS Statistics (version 21.0). ROC analysis, AUC, sensitivity, specificity, and 95% CIs calculation were also performed by IBM SPSS Statistics (version 21.0).

## 3 RESULTS

### 3.1 Isolation and characterization of fEVs

We isolated the EVs from faeces specimens by ultracentrifugation and conducted fundamental characterizations to verify their physical properties and chemical composition. The typical saucer‐shaped morphology of fEVs was directly observed via biological cryo‐transmission electron microscopy (cryo‐TEM) (Figure 1B). Nanoparticle tracking analysis (NTA) showed that the achieved fEVs had an average diameter of 152.6 ± 2.6 nm. Furthermore, western blotting was used to characterize the typical protein markers (Alix, CD9, and CD63) of EVs in the three randomly chosen faeces specimens (Figure 1C). These results proved that we have extracted fEVs out of faeces specimens successfully.

To examine whether the achieved fEVs were associated with CRC, western blotting was further performed to compare the expression signatures of CRC‐related proteins (typically CDH17, EGFR, EpCAM, HSP70, KRT19, and SRC) between the fEVs and CRC tissues. We isolated the fEVs from the faeces specimens of two CRC patients and collected their tumour tissues for western blotting analysis. As shown in Figure 1D, these proteins were detected in both fEVs and tumour tissues with good concordance, implying that the fEVs were highly linked to CRC tumours. We assumed that when the faeces reaches the near vicinity of CRC tumours, the tumour‐derived EVs could be carried by the faeces to naturally excrete from the intestinal tract. Thus, the fEVs could transport the unique molecular information of tumours outside the human body. Consequently, fEVs could reflect the pathologic condition of CRC lesions indirectly, providing a non‐invasive means to investigate the molecular status of CRC.

### 3.2 Screening protein biomarkers on fEVs for CRC diagnosis

**FIGURE 2:**
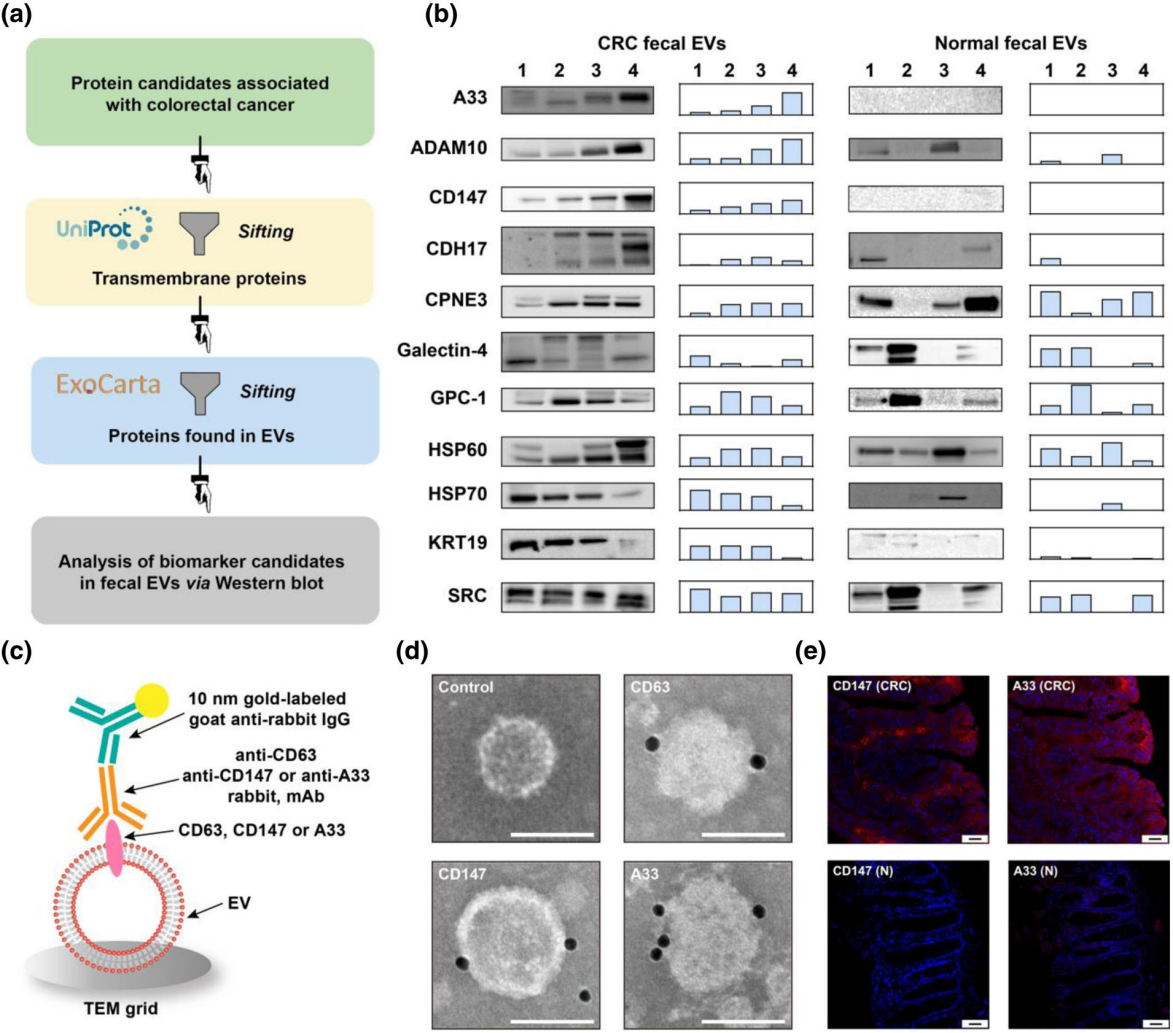
Screening of biomarker candidates and confirmation of CD147 and A33 on fEVs and tissues. (a) Procedures for screening biomarker candidates. (b) Western blotting analysis of fEVs from CRC patients and healthy donors (left) and the relative band intensity (right). (c) Schematic of immunogold labelling to confirm CD147 and A33 on fEVs. (d) Representative IG‐TEM pictures of CD63, CD147, and A33 labelled by 10 nm‐gold on fEVs. fEVs without primary antibody treatment were used as control. Scale bar: 100 nm. (e) Immunofluorescence (red) of CD147 and A33 in colorectal carcinoma (CRC) and para‐carcinoma (N) tissues. Scale bar: 50 μm

The prerequisite of using fEVs to diagnose CRC is to identify the proper fEV biomarkers. Accordingly, we conducted a screening procedure through integrating bioinformatics with the techniques of high‐throughput protein detection, as illustrated in Figure 2A. First, we employed bioinformatics to screen the CRC‐associated proteins that have been studied in previous reports. These proteins were then sifted with UniProt database (www.uniprot.org) to select the transmembrane ones, followed by ExoCarta database (www.exocarta.org) sifting for evaluating their presence on EVs. The detailed information on the biomarker candidates is presented in Supplementary Table S1.

Next, we performed western blotting to analyze the expression of these protein biomarker candidates on the membrane of fEVs. To do this, we collected the fEVs from CRC patients (*n =* 4) and healthy donors (*n =* 4) and compared their expression profiles of the biomarker candidates. The western blotting results (Figure 2B) showed that ADAM10, CDH17, CPNE3, Galectin‐4, GPC‐1, HSP60, HSP70, KRT19, and SRC on the fEVs were detected in both CRC and healthy groups. The relative band intensities showed the heterogeneity of protein expressions between the individuals. Excitingly, CD147 and A33 on the fEVs were exclusively found in the CRC group, indicating their high potential to be used as biomarkers for CRC diagnosis.

We confirmed the presence of CD147 and A33 on the surface of fEVs via immunogold transmission electron microscopy (IG‐TEM) (Figure 2C). Several gold nanoparticles were visible around the fEVs labelled with anti‐CD47, ‐A33, and ‐CD63 (a typical EV marker) immunogold tags independently, validating the expressions of CD147 and A33 on fEVs (Figure 2D).

Immunofluorescence staining was finally employed to determine whether CD147 and A33 are overexpressed in the CRC tissues. As shown in Figure 2E, the fluorescence intensity of CD147 and A33 in the CRC tissue was much higher than that of the adjacent healthy tissue. This result demonstrates that CRC tissues express CD147 and A33 at higher levels than the para‐cancer tissues, bridging the gap between fEVs and CRC tumours.

### 3.3 Construction of ELISA system to detect fEV proteins

**FIGURE 3:**
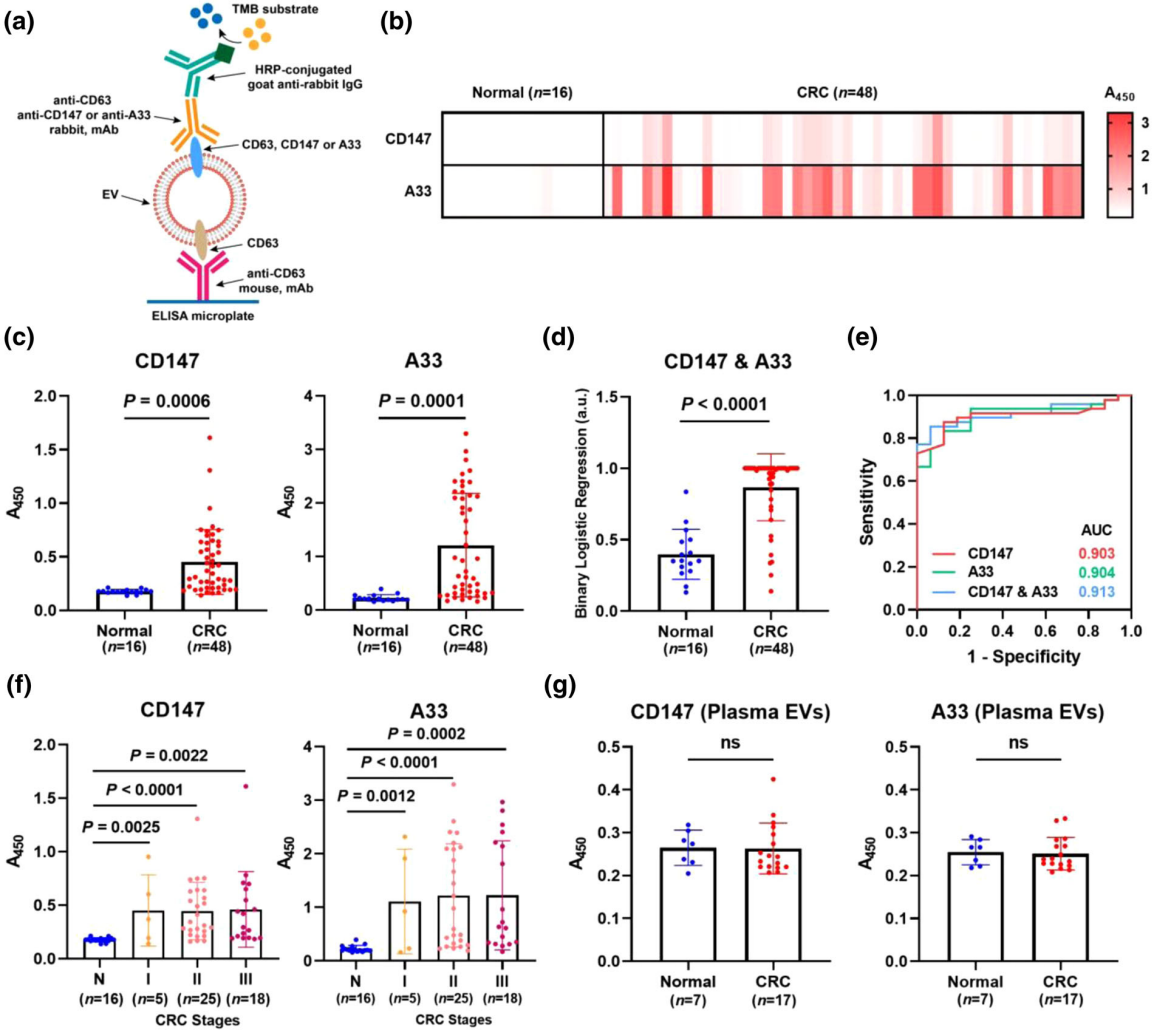
Analysis of CD147 and A33 in EVs from clinical faeces specimens. (a) Schematic of ELISA system to detect proteins on fEVs. Heat map (b) and scatter diagrams (c) of the CD147 and A33 levels on fEVs collected from healthy donors (*n* = 16) and CRC patients (*n* = 48). (d) The levels of CD147 and A33 were combined into a single index scatter plot by binary logistic regression analysis (CD147 & A33). (e) ROC curves of CD147, A33, and CD147 & A33 to differentiate CRC patients from healthy donors. (f) Levels of CD147 and A33 on fEVs across CRC stages. (g) Levels of CD147 and A33 in plasma‐derived EVs collected from healthy donors and CRC patients.

We performed an enzyme‐linked immunosorbent assay (ELISA) to detect proteins in fEVs (Figure 3A). Anti‐CD63 mouse antibody was used as the capture antibody, and anti‐CD63/CD147/A33 rabbit antibodies were used as the detection antibodies. HRP‐conjugated goat anti‐rabbit IgG was used as the secondary antibody. First, we used anti‐CD63 as the detection antibody to verify whether the ELISA construction was resultful. As shown in Supplementary Figure S2A, the detection signals of CD63 were proportional to the amounts of the added EVs, indicating that this ELISA method could efficiently quantify EV proteins. Next, we utilized this ELISA system to detect CD147 and A33 on the fEVs. The detection signal of CD147 went upward with the increase of EV concentration (Supplementary Figure S2B). Nevertheless, with the increase in EV amount, the detection signal of A33 rose at first and then dropped when the concentration of fEVs exceeded 40 μg/100 μl (Supplementary Figure S2C). This phenomenon probably results from the “high‐dose hook effect” when the antigen concentration exceeds the antibody concentration (Cavalera et al., 2022; Erickson & Grenache, 2016). Accordingly, the protein dosage for unified EV detection was set to be 40 μg/100 μl in the subsequent clinical sample detection and analysis to avoid this hook effect. Then, we optimized the detection antibody concentrations ranging from 0.25 to 2.0 μg/ml. According to the results in Supplementary Figure S2D and E, the optimal concentrations of the anti‐CD147 and anti‐A33 antibodies were determined to be 1.0 μg/ml and 2.0 μg/ml, respectively.

### 3.4 CRC diagnosis using CD147 and A33 on fEVs

The levels of CD147 and A33 on the fEVs of healthy donors ( *n =* 16) and CRC patients (*n =* 48) were detected by ELISA (Figure 3A). The clinical information of the healthy donors and CRC patients is shown in Supplementary Table S2. The heat map (Figure 3B) summarizes the expression signatures of CD147 and A33 in the normal and CRC groups, showing that the average levels of both markers were higher in CRC patients than in healthy donors. The scatter diagram (Figure 3C) exhibits statistical significance between the normal and CRC groups distinguished by CD147 (*P =* 0.0006, unpaired *t*‐test) and A33 (*P =* 0.0001, unpaired *t*‐test). Moreover, CD147 and A33 were combined into a complementary marker (CD147 & A33) by binary logistic regression analysis to distinguish CRC from the normal group (*P <* 0.0001, unpaired *t*‐test) (Figure 3D). Furthermore, using receiver operating characteristic curve (ROC) analysis (Figure 3E), the area under curve (AUC) values were calculated to investigate the diagnostic efficiency of CD147 and A33. The ROC analysis results (Supplementary Table S3) showed that both CD147 (AUC = 0.903) and A33 (AUC = 0.904) were effective in differentiating the CRC patients from the healthy donors. The combination of CD147 and A33 showed a higher AUC of 0.913.

To examine whether the levels of CD147 and A33 on fEVs are associated with the stages of CRC, the patients were stratified by TNM staging. Of the 48 patients with CRC, the levels of CD147 and A33 did not vary significantly across the stages. However, when the individual stage was compared with the healthy group, significant differences were obtained (unpaired *t*‐test), as illustrated in Figure 3F.

We next investigated whether CD147 and A33 can be detected in the plasma EVs of healthy donors (*n =* 7) and patients with CRC (*n =* 17). As shown in Figure 3G, the levels of CD147 and A33 in plasma EVs were statistically identical between the healthy and CRC groups (*P>*0.05), indicating that fEVs are more pathologically relevant to CRC than plasma EVs. Since blood circulates throughout the body, plasma contains numerous EVs derived from different cells and tissues (Alberro et al., 2021). In contrast, fEVs closely correlate with the intestinal tract and therefore contain distinct physiological signatures of the intestinal environment. Accordingly, fEVs are more associated with CRC lesions than plasma EVs.

In addition, we compared the fEV biomarkers with the clinically used serum marker CEA in 35 CRC patients. CEA, CD147, A33, and CD147 & A33 were used to diagnose CRC individually. The cut‐off value of CEA in the blood is 5 ng/ml, while the cut‐off values for CD147, A33, and CD147 & A33 are set to be 0.192, 0.277, and 0.625 according to the ROC analysis results, respectively. As shown in Supplementary Figure S3, the positive and negative results were marked in red and green, respectively. Obviously, CD147, A33, and CD147 & A33 (all 31 out of 35, 89%) outperformed CEA (14 out of 35, 40%) in terms of diagnostic sensitivity.

### 3.5 Analysis of longitudinal faeces specimens from CRC patients

**FIGURE 4:**
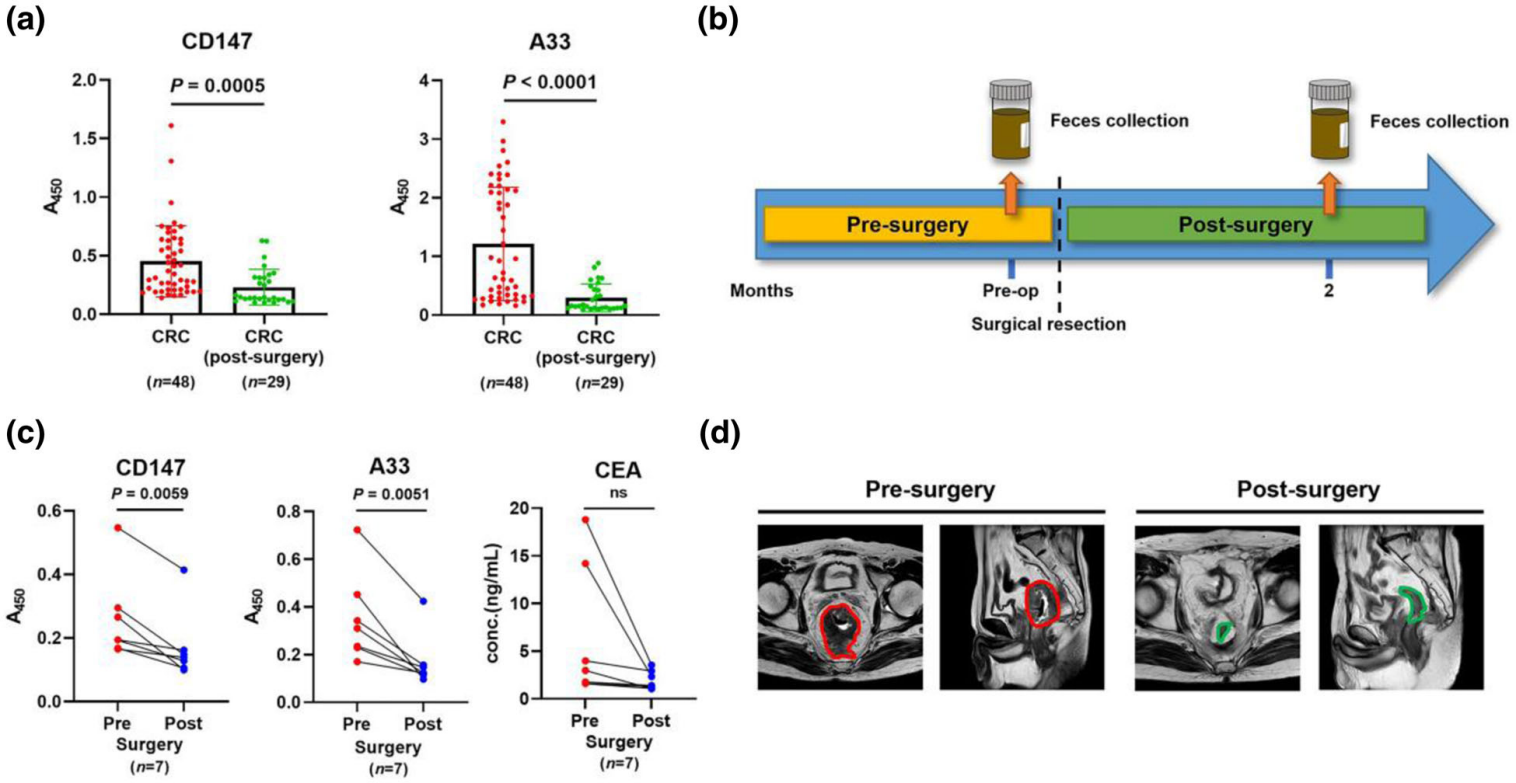
Analysis of CD147 and A33 in a longitudinal study. (a) Levels of CD147 and A33 on fEVs from CRC patients before and after surgery. (b) Longitudinal faeces collection preoperatively and postoperatively (month 2). (c) Analysis of longitudinal faeces specimens from seven CRC patients who underwent surgery. (d) Representative MRI of a CRC patient before and after surgery, with tumour and post‐resection site encircled in red and green, respectively.

An ideal biomarker should function as both a high‐efficacy diagnostic indicator and a prognostic reporter to monitor the effectiveness of clinical interventions. ELISA was used to analyze the levels of CD147 and A33 on the fEVs of preoperative (*n =* 48) and postoperative (*n =* 29) CRC patients, aiming at assessing the possibility of employing these markers as prognostic reporters. As shown in Figure 4A, the average levels of CD147 and A33 were significantly lower in the patients after surgery (*P =* 0.0005 for CD147 and *P* < 0.0001 for A33, unpaired *t*‐test). Then, we tracked the changes of fEV biomarkers in the same CRC patients before and after the clinical surgery. Faeces specimens (*n =* 7) were collected preoperatively and 2 months after surgery (Figure 4B). The levels of CD147 and A33 on fEVs decreased in all seven individuals (*P =* 0.0059 for CD147 and *P =* 0.0051 for A33, paired *t*‐test). Although CEA levels in the serum of seven patients also decreased after surgery, the overall downward trend was not statistically significant (*P =* 0.11, paired *t*‐test) (Figure 4C). Magnetic resonance imaging (MRI) of a CRC patient before and after surgery was shown in Figure 4D, demonstrating that the decrease in biomarker levels was associated with tumour resection.

## 4 DISCUSSION

CRC is one of the most common malignancies worldwide, posing a severe threat to human health. Early diagnosis of CRC can significantly improve the 5‐year survival rate of patients. Faeces is naturally excreted through normal physiological processes and is strongly linked to the intestinal environment, making it a readily available sample and an ideal analyte for CRC screening in a non‐invasive manner. However, current CRC screening methods based on faeces samples (such as FIT and mstDNA test) are still limited by their low clinical sensitivity and specificity. To tackle this challenge, effective faecal biomarkers are urgently needed for early CRC diagnosis.

In this study, we identified two biomarkers on fEVs for CRC detection, CD147 and A33. CD147 is expressed in the tissues of various malignancies (Li et al., 2009; Riethdorf et al., 2006). It is reported that CD147/CD9 double‐positive EVs (Yoshioka et al., 2014) and CD147‐positive EVs (Tian et al., 2018) in blood samples could serve as CRC biomarkers. A33 is specifically positive in the tumours of the gastrointestinal tract. A33 is also a cell surface target for antibody‐based therapy (GarinChesa et al., 1996). The level of A33 in plasma EVs was evaluated before; when differentiating between CRC patients and healthy individuals, A33 displayed limited diagnostic efficiency, with an AUC value of 0.65 and a sensitivity of 47% (Park et al., 2021). CRC tumours are situated in the intestinal tract; therefore, biological signalling molecules released by the CRC lesions are prone to enter the intestinal environment. These physiological and pathological signatures were brought out by faeces through natural defecation, making it possible to conduct CRC‐related profiling in vitro. Accordingly, fEVs could provide more direct and accurate bio‐information concerning CRC than plasma EVs. Based on these rationales, we turned our attention to fEVs and investigated their correlation with CRC.

To our knowledge, this is the first study that proceeded from fEVs to find biomarkers for CRC. We discovered that CD147 and A33 on fEVs could distinguish CRC patients from healthy donors with AUC values of 0.903 and 0.904, respectively. Moreover, their combination enabled by logistic regression could further improve the clinical accuracy (AUC = 0.913). The biomarkers CD147 and A33 on fEVs outperform those in plasma EVs and serum CEA regarding diagnostic accuracy, compliance, and simplicity. Besides, we proved that CD147 and A33 on fEVs had the potential to monitor the prognosis of CRC. These findings are expected to prompt the development of clinical CRC screening and prognosis monitoring.

Despite these promising findings, there are still several limitations in this study. First, CD147 and A33 on fEVs were unable to distinguish different CRC stages, although significant differences were obtained when comparing healthy donors with each stage of CRC. The possible reason could be ascribed to the limited sensitivity of the ELISA system. Furthermore, tumours themselves display complex diversity and heterogeneity at different stages. Second, the analysis of fEVs relies on isolation via pretreatment and ultracentrifugation of faeces samples, making it a time‐consuming and complicated procedure. To solve this problem, it is urgently needed to develop integrated isolation‐detection platforms with high sensitivity in the future. Third, the patient cohort in our study (16 healthy donors, 48 CRC patients, and 29 CRC patients after surgery) is not large enough; further investigations in a larger‐scale cohort are required to explore the clinical utility of CD147 and A33 on fEVs for CRC screening.

Gram‐positive and negative bacteria are essential members of the intestinal environment, which release bacterial extracellular vesicles (BEVs) through different formation routes (Brown et al., 2015; Schwechheimer & Kuehn, 2015; Toyofuku et al., 2019). Accumulating evidence has proved that fEVs are a collection of BEVs and host‐derived eukaryotic EVs (Northrop‐Albrecht et al., 2022; Tulkens et al., 2020). At present, the separation of subpopulations in fEVs is still dependent on ultrafiltration (UF) followed by density gradient centrifugation and size‐exclusion chromatography (SEC), making it a labour‐intensive and time‐consuming procedure (Kameli et al., 2021; Tulkens et al., 2020). Accordingly, it is significant to develop approaches to separate these two EVs populations in faeces samples, which is expected to facilitate the identification of CRC biomarkers in fEVs with enhanced sensitivity.

Apart from EV protein, RNA is another vital aspect of EV candidate biomarkers. With the assistance of RNA sequencing (RNA‐Seq) and quantitative reverse transcription PCR (RT‐qPCR), RNA biomarkers are of increasing interest and are in rapid development (He et al., 2020; Liu et al., 2022; Min et al., 2019; Ostenfeld et al., 2016; Wang et al., 2022). Although faecal RNAs have been revealed as biomarkers for CRC (Duran‐Sanchon et al., 2020; Herring et al., 2021; Wu et al., 2012; Yau et al., 2014), few have concentrated on RNAs in isolated fEVs. Hence, RNA biomarkers for CRC in fEVs remain to be further investigated.

In conclusion, our work shows that fEVs can be used as new biomarkers for CRC diagnosis and prognosis with high clinical sensitivity and sensitivity, providing new opportunities for mass CRC screening in an entirely non‐invasive manner. This discovery fills the gap between EVs and clinical diagnosis, which will prompt the applications of fEVs in both fundamental research and clinical diagnosis of CRC.

## AUTHOR CONTRIBUTIONS

Zhaowei Zhang: Data curation; Formal analysis; Methodology; Project administration; Writing—original draft. Xuehui Liu: Data curation; Methodology; Project administration; Software. Xiaoqing Yang: Data curation; Formal analysis; Validation; Writing—original draft. Ying Jiang: Data curation; Methodology; Resources; Visualization. Ang Li: Data curation; Methodology; Resources. Jiying Cong: Data curation; Validation. Yuwei Li: Data curation; Formal analysis; Validation. Qinjian Xie: Data curation; Resources; Validation. Chen Xu: Conceptualization; Formal analysis; Resources; Writing—review & editing. Dingbin Liu: Conceptualization; Formal analysis; Funding acquisition; Investigation; Resources; Supervision; Writing—review & editing

## CONFLICTS OF INTEREST

All the authors declare no competing interests.

## ETHICAL STATEMENT

This study was approved by the Ethics Committee of Tianjin People's Hospital (No. 2021‐B12). All experiments using clinical samples were conducted under relevant ethical guidelines, and all participants received written informed consent.

## Supporting information

Supplementary information

## Data Availability

All data are available in the main text or the supplementary material, or are available from the corresponding author on reasonable request.
