# Supplementary material for: Identification of faecal extracellular vesicles as novel biomarkers for the non‐invasive diagnosis and prognosis of colorectal cancer
Source: J Extracell Vesicles. 2023 Jan 5;12(1):12300. doi: 10.1002/jev2.12300 (PMC9816085; doi:10.1002/jev2.12300)
Supplement: Supplementary file 1 — Supplementary information [file JEV2-12-12300-s001.docx]

*Supplementary Material* for

**Identification of faecal extracellular vesicles as novel biomarkers for the non-invasive diagnosis and prognosis of colorectal cancer**

Zhaowei Zhang,^†^ Xuehui Liu,^†^ Xiaoqing Yang,^†^ Ying Jiang, Ang Li, Jiying Cong, Yuwei Li, Qinjian Xie, Chen Xu,^*^ Dingbin Liu^*^

*Corresponding authors. Email: liudb@nankai.edu.cn; xc198129@163.com

†These authors contributed equally to this work.


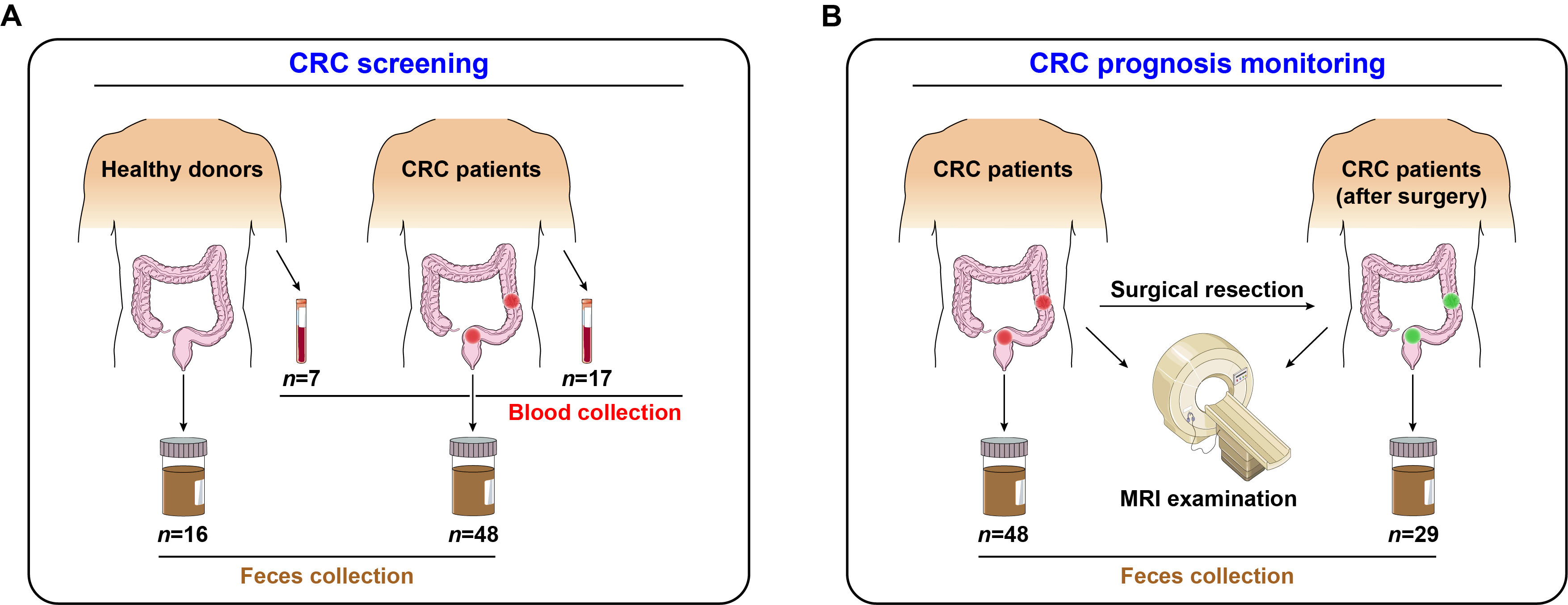


**Supplementary Fig. S1. Workflow of CRC screening and prognosis monitoring.** (**A**) CRC screening: blood and feces specimens were collected from healthy donors and CRC patients. (**B**) CRC prognosis monitoring: feces specimens were collected from CRC patients and CRC patients who underwent surgical resection.


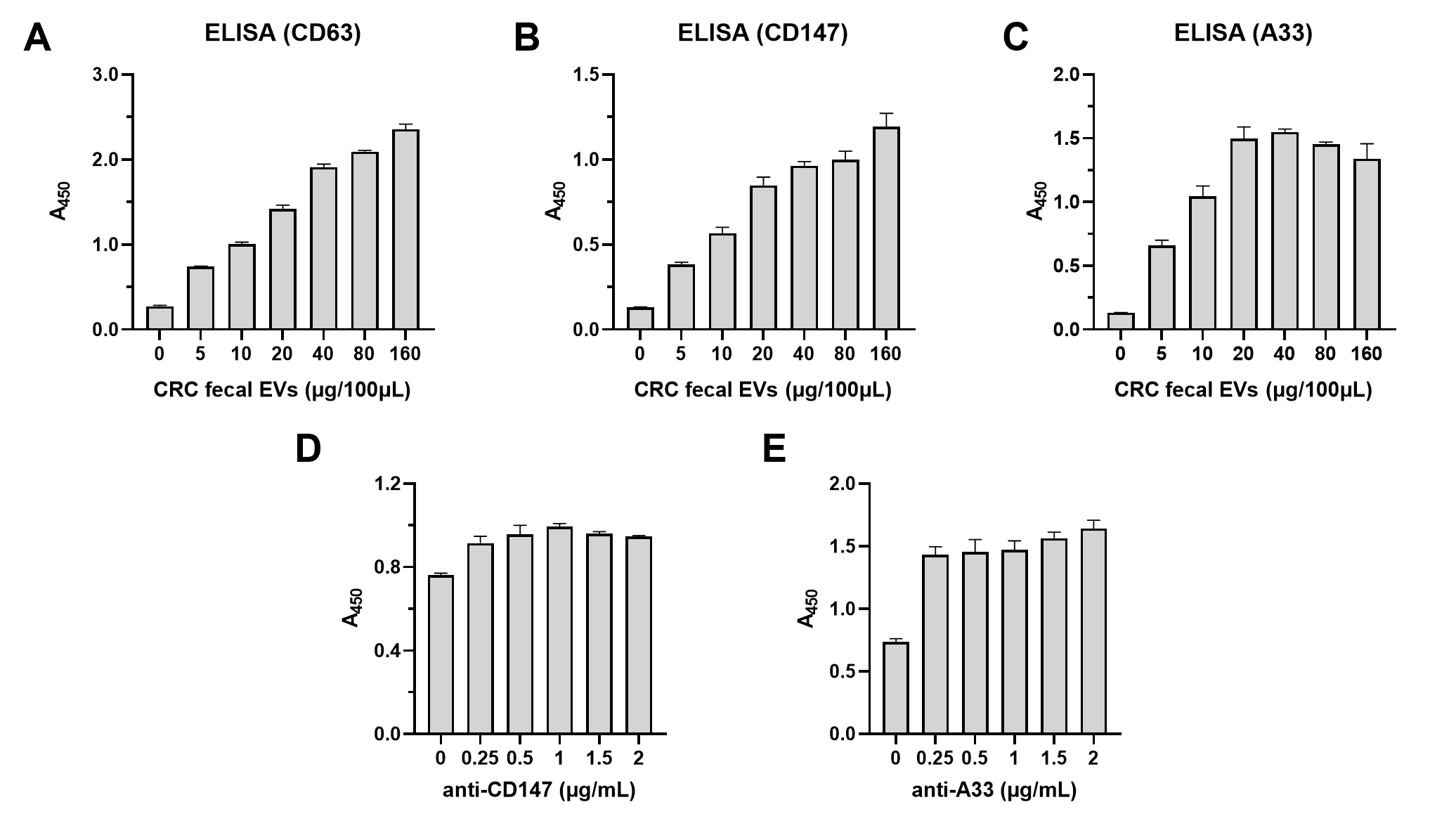


**Supplementary Fig. S2. ELISA construction and detection of fecal EV proteins.** (**A**) The expression of CD63 in different concentrations of fEVs by ELISA. (**B**) The expression of CD147 in different concentrations of fEVs by ELISA. (**C**) The expression of A33 in different concentrations of fEVs by ELISA. (**D**) and (**E**) Optimization of CD147 and A33 antibody concentrations.


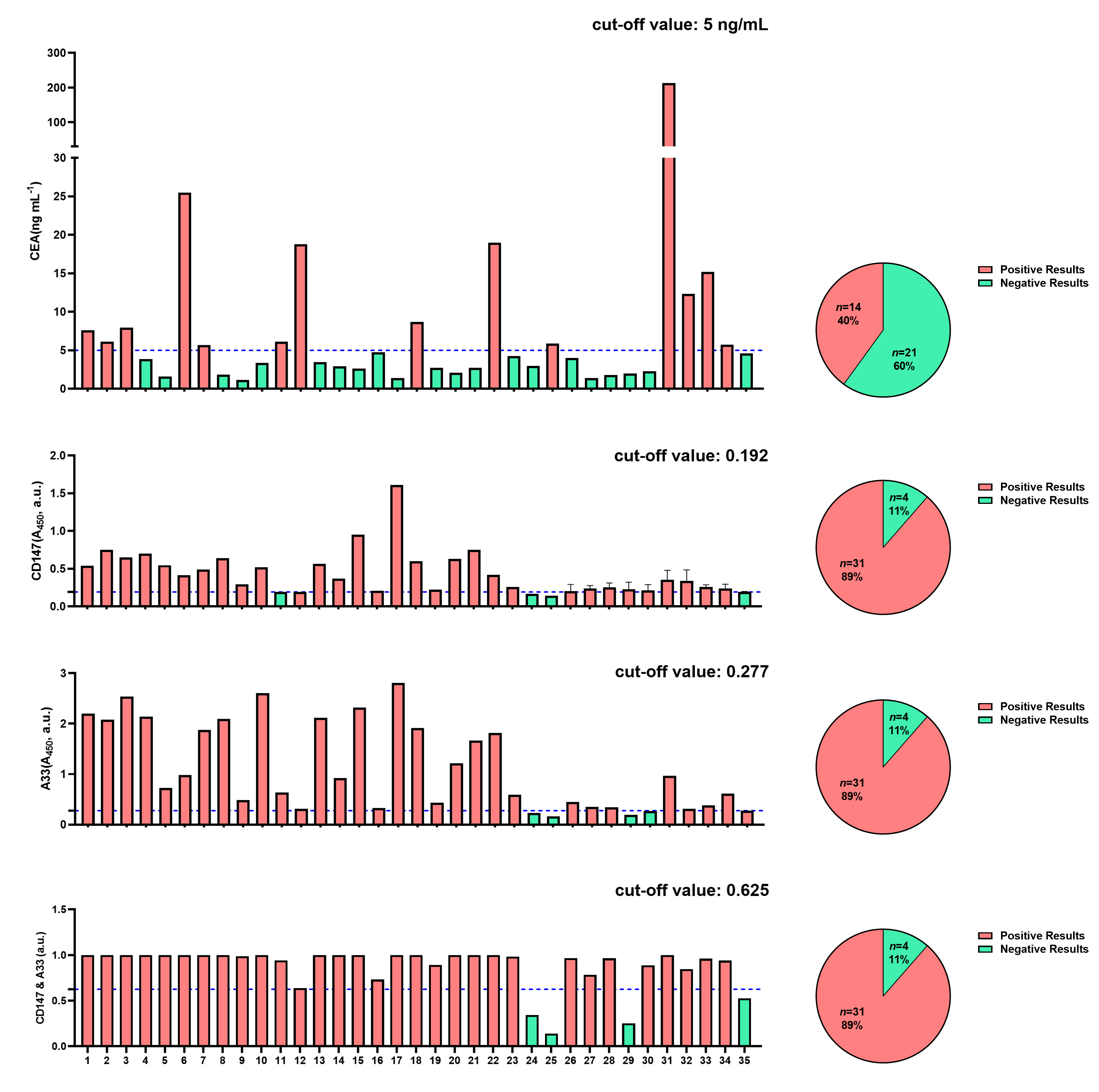


**Supplementary Fig. S3. Comparison of serum CEA, fecal EV-CD147, fecal EV-A33 and CD147 & A33 by binary logistic regression, of 35 CRC patients.**

**Supplementary Table S1. Biomarker candidates for CRC detection.**

| **Protein Name** | **Descriptions** | **References** |
| --- | --- | --- |
| Glycoprotein A33  (A33) | A glycoprotein biomarker that is over-expressed by CRC. | 1 |
| ADAM metallopeptidase domain 10  (ADAM10) | Correlates with CRC malignancy and promotes CRC cell migration and invasion. | 2 |
| Transmembrane protein 147  (CD147) | The concentration of CD147-positive EVs in plasma of CRC patients is significantly increased. | 3 |
| Cadherin-17  (CDH17) | Overexpressed in CRC and has been proposed as a disease marker for hepatic and gastrointestinal malignancies. | 4 |
| Copine III  (CPNE3) | A biomarker in plasma-derived exosomes for the diagnosis and prognosis of CRC. | 5 |
| Galectin-4 | Functions as a tumor suppressor, and its loss is an important event in CRC tumorigenesis. | 6 |
| Glypican 1  (GPC-1) | The increased plasma GPC1^+^ exosomes are specific markers for the diagnosis of CRC. | 7 |
| Heat shock proteins  (HSP60 and HSP70) | HSP60 has potential as a biomarker in patients with large bowel cancer.  HSP70 can serve as a risk indicator in CRC. | 8, 9 |
| Keratin 19  (KRT19) | Correlates with colorectal tissue. | 10 |
| SRC proto-oncogene  (SRC) | 80% of patients with colon cancer overexpress SRC in tumor tissue. | 11 |

**Supplementary Table S2. Clinical information of healthy donors and CRC patients.**

|  | Feces | | | Plasma | |
| --- | --- | --- | --- | --- | --- |
| Characteristic | Patients with CRC  (*n=*48) | Healthy Donors  (*n=*16) | Patients with CRC post-surgery  (*n=*29) | Patients with CRC  (*n=*17) | Healthy  Donors  (*n=*7) |
| Age (years) |  |  |  |  |  |
| Mean | 63 | 48 | 63 | 66 | 42 |
| Median  (range) | 64.5  (39-83) | 50.5  (27-72) | 66  (34-75) | 67  (52-79) | 33  (24-65) |
| Gender |  |  |  |  |  |
| Male | *n=*32 | *n=*12 | *n=*18 | *n=*8 | *n=*2 |
| Female | *n=*16 | *n=*4 | *n=*11 | *n=*9 | *n=*5 |
| Stage |  |  |  |  |  |
| I | *n=*5 | — | — | — | — |
| II | *n=*25 | — | — | — | — |
| III | *n=*18 | — | — | — | — |

**Supplementary Table S3. ROC analysis of CD147, A33, and CD147 & A33 to differentiate between healthy donors and CRC patients.**

|  | AUC | 95%CI | Sensitivity | Specificity |
| --- | --- | --- | --- | --- |
| CD147 | 0.903 | 0.803-0.963 | 0.875 | 0.875 |
| A33 | 0.904 | 0.805-0.964 | 0.833 | 0.875 |
| CD147 & A33 | 0.913 | 0.815-0.969 | 0.854 | 0.937 |

**Supplementary Table S4.** **Detailed information of antibodies.**

| **Antibody Name** | **Source** | **Identifier** |
| --- | --- | --- |
| Anti-CD9  (Rabbit, pAb) | Beyotime Biotechnology  (Shanghai, China) | Cat. #AF0108 |
| Anti-Alix  (Mouse, mAb) | Abcam  (Cambridge, MA) | Cat. #ab117600 |
| Anti-CD63  (Rabbit, pAb) | Proteintech  (Chicago, USA) | Cat. #25682-1-AP |
| Anti-CD63  (Mouse, mAb) | SinoBiological  (Beijing, China) | Cat. #11271-MM10 |
| Anti-A33  (Rabbit, mAb) | SinoBiological  (Beijing, China) | Cat. #11277-R071 |
| Anti-ADAM10  (Rabbit, pAb) | Proteintech  (Chicago, USA) | Cat. #25900-1-AP |
| Anti-CD147  (Rabbit, mAb) | SinoBiological  (Beijing, China) | Cat. #10186-R118 |
| Anti-CDH17  (Rabbit, pAb) | Proteintech  (Chicago, USA) | Cat. #24339-1-AP |
| Anti-CPNE3  (Rabbit, pAb) | Proteintech  (Chicago, USA) | Cat. #11186-1-AP |
| Anti-EGFR  (Rabbit, pAb) | SinoBiological  (Beijing, China) | Cat. #10001-RP01 |
| Anti-EpCAM  (Mouse, mAb) | SinoBiological  (Beijing, China) | Cat. #10694-MM05 |
| Anti-Galectin-4  (Rabbit, pAb) | Proteintech  (Chicago, USA) | Cat. #27552-1-AP |
| Anti-GPC-1  (Rabbit, pAb) | Proteintech  (Chicago, USA) | Cat. #16700-1-AP |
| Anti-HSP60  (Rabbit, pAb) | Proteintech  (Chicago, USA) | Cat. #15282-1-AP |
| Anti-HSP70  (Rabbit, pAb) | Proteintech  (Chicago, USA) | Cat. #10995-1-AP |
| Anti-KRT19  (Rabbit, pAb) | Proteintech  (Chicago, USA) | Cat. #10712-1-AP |
| Anti-SRC  (Rabbit, pAb) | Proteintech  (Chicago, USA) | Cat. #11097-1-AP |
| PE-conjugated Anti-A33  (Rabbit, mAb) | SinoBiological  (Beijing, China) | Cat. #11277-R074-P |
| PE-conjugated Anti-CD147  (Rabbit, mAb) | SinoBiological  (Beijing, China) | Cat. #10186-R125-P |
| 10 nm-gold labeled  Goat Anti-Rabbit IgG H&L | Bioss  (Beijing, China) | Cat. #bs-0295G-Gold |
| HRP-labeled  Goat Anti-Rabbit IgG (H+L) | Beyotime Biotechnology (Shanghai, China) | Cat. #A0208 |
| HRP-labeled  Goat Anti-Mouse IgG (H+L) | Beyotime Biotechnology (Shanghai, China) | Cat. #A0216 |

pAb, polyclonal antibody; mAb, monoclonal antibody.

**References**

1. Adumeau, P. et al. A Pretargeted Approach for the Multimodal PET/NIRF Imaging of Colorectal Cancer. *Theranostics* **6**, 2267-2277 (2016).

2. Sun, L. et al. Epigenetic Regulation of a Disintegrin and Metalloproteinase (ADAM) Transcription in Colorectal Cancer Cells: Involvement of beta-Catenin, BRG1, and KDM4. *Front Cell Dev. Biol.* **8**, 581692 (2020).

3. Tian, Y. et al. Protein Profiling and Sizing of Extracellular Vesicles from Colorectal Cancer Patients via Flow Cytometry. *ACS Nano* **12**, 671-680 (2018).

4. Bartolome, R. A. et al. Cadherin-17 interacts with alpha2beta1 integrin to regulate cell proliferation and adhesion in colorectal cancer cells causing liver metastasis. *Oncogene* **33**, 1658-1669 (2014).

5. Sun, B. et al. Circulating exosomal CPNE3 as a diagnostic and prognostic biomarker for colorectal cancer. *J. Cell Physiol.* **234**, 1416-1425 (2019).

6. Satelli, A., Rao, P. S., Thirumala, S. & Rao, U. S. Galectin-4 functions as a tumor suppressor of human colorectal cancer. *Int. J. Cancer* **129**, 799-809 (2011).

7. Li, J. et al. GPC1 exosome and its regulatory miRNAs are specific markers for the detection and target therapy of colorectal cancer. *J. Cell Mol. Med.* **21**, 838-847 (2017).

8. Campanella, C. et al. Heat shock protein 60 levels in tissue and circulating exosomes in human large bowel cancer before and after ablative surgery. *Cancer* **121**, 3230-3239 (2015).

9. Jubran, R. et al. Circulating mitochondrial stress 70 protein/mortalin and cytosolic Hsp70 in blood: Risk indicators in colorectal cancer. *Int. J. Cancer* **141**, 2329-2335 (2017).

10. Xiao, Y. et al. The potential of exosomes derived from colorectal cancer as a biomarker. *Clin. Chim. Acta* **490**, 186-193 (2019).

11. Chen, J., Elfiky, A., Han, M., Chen, C. & Saif, M. W. The role of Src in colon cancer and its therapeutic implications. *Clin. Colorectal Cancer* **13**, 5-13 (2014).
